# Saunders’ Medical Hand Atlases

**Published:** 1901-07

**Authors:** 


SAUNDERS' MEDICAL HAND ATLASES.—ATLAS AND EPITOME OF THE NERVOUS SYSTEM AND ITS DISEASES. By Prof. Dr. Chr. Jakob, of Erlangen. From the second revised German edition. Edited by Edward D. Fisher, M. D., Professor of Diseases of the Nervous System, University and Bellevue Medical College, N. Y. With 83 plates and copious text. Philadelphia and London: W. B. Saunders & Co. 1901. Cloth, $3.50 net.

All that can be done by text and illustrations to make the peculiarities of the normal and pathologic anatomy of the nervous system intelligible to the student is accomplished by the subject matter of this excellent work. In the preparation of the explanatory text all unnecessary details have been omitted.

The illustrations represent actual conditions. They are, therefore, not mere diagramatic representations. Too much cannot be said in praise of the work done on the 83 lithographic plates.

